# Can individual functional improvements be predicted in osteoarthritic patients after total knee arthroplasty?

**DOI:** 10.1186/s43019-024-00238-1

**Published:** 2024-10-14

**Authors:** Sung Eun Kim, Du Hyun Ro, Myung Chul Lee, Hyuk-Soo Han

**Affiliations:** 1 Department of Orthopaedic Surgery, Seoul National University College of Medicine, Seoul, South Korea; 2 Department of Orthopaedic Surgery, Seoul National University Hospital, 101 Daehak-Ro, Jongno-Gu, Seoul, 110-744 South Korea; 3 CONNECTEVE Co. Ltd., Seoul, South Korea; 4 Innovative Medical Technology Research Institute, Seoul National University Hospital, Seoul, South Korea; 5 SNU Seoul Hospital, Seoul, South Korea

**Keywords:** WOMAC, Knee arthroplasty, Predictive model, Functional improvement

## Abstract

**Purpose:**

Total knee arthroplasty (TKA) is an effective treatment for advanced osteoarthritis, and achieving optimal outcomes can be challenging due to various influencing factors. Previous research has focused on identifying factors that affect postoperative functional outcomes. However, there is a paucity of studies predicting individual postoperative improvement following TKA. Therefore, a quantitative prediction model for individual patient outcomes is necessary.

**Materials and methods:**

Demographic data, radiologic variables, intraoperative variables, and physical examination findings were collected from 976 patients undergoing TKA. Preoperative and 1-year postoperative Western Ontario and McMaster Universities Osteoarthritis Index (WOMAC) scores were assessed, and multivariate regression analysis was conducted to identify significant factors influencing one-year WOMAC scores and changes in WOMAC scores. A predictive model was developed on the basis of the findings.

**Results:**

The predictive accuracy of the model for 1-year WOMAC scores was poor (all adjusted *R*^2^ < 0.08), whereas the model for changes in WOMAC scores demonstrated strong predictability (all adjusted *R*^2^ > 0.75). Preoperative WOMAC scores, sex, and postoperative knee range of motion significantly affected all pain, stiffness, and physical function aspects of the WOMAC scores (all *P* < 0.05). Age, cerebrovascular disease, and patellar resurfacing were associated with changes in physical function (all *P* < 0.05).

**Conclusions:**

The developed quantitative model demonstrated high accuracy in predicting changes in WOMAC scores after TKA. The identified factors influencing postoperative improvement in WOMAC scores can assist in optimizing patient outcomes after TKA.

**Supplementary Information:**

The online version contains supplementary material available at 10.1186/s43019-024-00238-1.

## Introduction

Total knee arthroplasty (TKA) is a widely performed and effective treatment for patients with advanced osteoarthritis, leading to positive and satisfactory outcomes [1]. However, defining what constitutes an optimal result can be subjective, as patients may still experience discomfort, functional impairment, and dissatisfaction even if the surgery is technically successful according to the surgeon’s assessment [2]. Thus, patient-reported outcome measures (PROMs) have been developed to address the patient’s perspective on outcomes [3]. These measures include subjective outcome assessments by patients and are widely used in clinical practice to evaluate the effectiveness of surgery in daily function. Among the various PROMs, the Western Ontario and McMaster Universities Osteoarthritis Index (WOMAC), is a widely used tool to evaluate PROMs after TKA. And its validity, reliability, and responsiveness have been well established [4].

The TKA outcomes are influenced by various factors, including patient-related and surgical factors [5]. The interaction of these factors plays a significant role in determining both patient function and overall outcomes. Therefore, considering these factors is crucial to achieving optimal outcomes after TKA as they provide valuable information about when and under what circumstances the optimal results can be achieved. However, an optimal outcome may have two aspects: achieving the best possible clinical score and attaining maximal improvement after surgery [6].

Prior research on this issue was limited to the qualitative identification of factors with a dichotomous impact on PROMs [7]. However, there are few studies that quantitatively predicted individual patient outcomes [8], such as determining the PROMs after TKA at a certain time. Therefore, the primary objective of this study was to develop a model that can quantitatively predict the outcome of an individual patient. To achieve this, we evaluated the factors contributing to the best WOMAC scores and the most significant improvement in WOMAC scores following TKA.

## Materials and methods

**Fig. 1:**
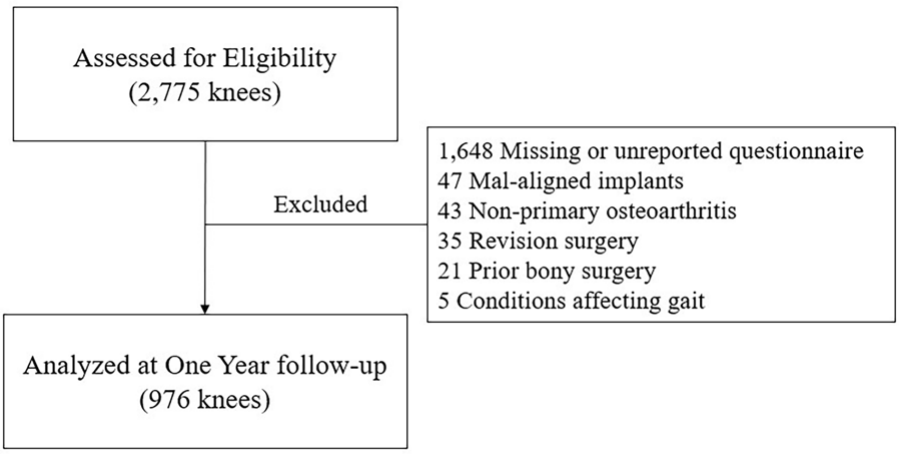
Flowchart of the study

With the approval of the institutional review board (H-2304-001-1416), a retrospective analysis of electronic medical records for patients who underwent primary TKA for knee osteoarthritis at the author’s institute between January 2002 and January 2021 was performed. The study included patients with a minimum 1-year follow-up period who had completed WOMAC questionnaires. Exclusion criteria included patients with non-primary osteoarthritis, such as those with rheumatoid arthritis or posttraumatic arthritis. Additionally, patients with conditions affecting gait, a history of prior bony surgery, or those who underwent revision surgery during the follow-up period owing to aseptic loosening, instability, dislocation, postoperative infection, or periprosthetic fractures were excluded from the analysis, as these conditions are known to negatively affect PROMs[9]. The flowchart of patients’ knees included in this study is presented in Fig. 1.

### Data collection

Demographic factors and comorbidities (e.g., cerebrovascular disease, diabetes mellitus, hypertension, ischemic heart disease, chronic liver disease, and chronic renal disease) were collected. Cerebrovascular disease was defined as a history of stroke or transient ischemic attack without residual hemiparesis or hemiplegia. Radiologic variables were assessed both preoperatively and 1 year after surgery, including the hip-knee-ankle angle (HKAA) to evaluate coronal plane deformity, and the Blackburne–Peel ratio to assess joint line, patellar height, and the posterior tibial slope [10]. Patients with a Blackburne-Peel ratio below 0.54 or malaligned implants (a distal femoral angle deviating by more than 3° from 5° valgus, a posterior tibial angle deviating by more than 3° from the mechanical axis, and a posterior tibial slope outside the 0°–5° range), which are both poor predictors of PROMs, were excluded from the analysis[11, 12]. The radiologic measurements were performed by two orthopedic specialists specializing in knee surgery. Physical examination findings, such as knee flexion contracture (inability to fully extend the knee) and the knee flexion angle, were recorded preoperatively and 1 year after surgery. WOMAC scores, including subcategories of pain, stiffness, and physical function, were collected preoperatively and 1 year after TKA. These subcategories were scored on scales of 0–20 for pain, 0–8 for stiffness, and 0–68 for physical function, with higher scores denoting poorer outcomes [4]. The change ($\Delta )$ in WOMAC scores was calculated by subtracting the preoperative WOMAC score from the postoperative score at the one-year follow-up. Table 1 presents the demographic characteristics of the patients, while Table 2 displays the preoperative, one-year, and $\Delta$ WOMAC scores.

**Table 1:** Demographic characteristics

*N* = 976	Mean ± SD	Range
Age (years)	74.7 ± 7.1	50 to 95
Sex (female)	94.4%	
Body mass index	26.7 ± 3.3	15.8 to 38.1
Preoperative HKAA*	9.9 ± 5.7	−21.3 to 30.8
Postoperative HKAA*	1.6 ± 2.1	−6.7 to 12.3
Cerebrovascular disease	10.5%	
Diabetes mellitus	26.7%	
Hypertension	38.1%	
Ischemic heart disease	10.7%	
Chronic liver disease	3.8%	
Chronic kidney disease	3.4%	
Patella resurfacing	66.1%	
Preoperative flexion contracture^†^	9.2 ± 7.7	−5 to 60
Preoperative flexion angle	124.7 ± 15.9	45 to 150
Postoperative flexion contracture^†^	0.4 ± 2.3	−5 to 20
Postoperative flexion angle	127.6 ± 10.6	70 to 150

^*^Positive values indicate varus, while negative values indicate valgus

^†^Negative values indicate recurvatum

Categorical variables are presented as percentages (%)

Continuous variables are presented as means ± standard deviations

*HKAA* Hip-knee-ankle angle, *SD* Standard deviation

**Table 2:** Preoperative, 1-year, and change ($\Delta )$ in WOMAC scores

	Preoperative	1-Year	Change ($\Delta )$*
WOMAC pain	9.3 ± 3.4 (1 to 20)	0.6 ± 1.2 (0 to 7)	−8.6 ± 3.1 (− 20 to 0)
WOMAC stiffness	3.9 ± 2.0 (0 to 8)	0.9 ± 1.1 (0 to 5)	−3.0 ± 1.9 (-8 to 5)
WOMAC physical function	35.3 ± 12.5 (5 to 68)	10.0 ± 6.8 (0 to 44)	−25 ± 12.0 (−67 to 10)

^*^Negative values indicate clinical improvement

$\boldsymbol{\Delta }$, Change in WOMAC scores; *WOMAC* Western Ontario and McMaster Universities Osteoarthritis Index

### Perioperative protocols

All TKA procedures were performed by a single surgeon over 20 years of experience in knee surgery in a tertiary hospital. A parapatellar arthrotomy was performed, followed by the excision of both cruciate ligaments. The modified gap balancing technique was utilized to achieve a neutral mechanical alignment targeting a neutral HKAA during bone cuts. Posterior stabilized and fixed implants were used. The decision for patellar resurfacing or preservation was based on the International Cartilage Repair Society (ICRS) grade of the patellar articular surface and was documented intraoperatively. Resurfacing was performed for a grade of 3 or higher, whereas preservation was preferred for patellae with a thickness below 20 mm to reduce the risk of patellar fracture[13]. All implants were fixed with cement.

Standardized rehabilitation protocols were implemented for all patients. Early postoperative ambulation and continuous passive motion exercises started on the day following surgery. Physical therapists began gentle passive range of motion (ROM) exercises three days postoperatively. Follow-up visits were scheduled at 1 month, 3 months, 12 months, and annually thereafter.

### Statistical analysis

Multivariate regression analysis using the ordinary least squares method was conducted to identify variables affecting the one-year and ΔWOMAC scores. Variables were selected through univariate analysis with a significance level of 20%, and then multivariate analysis was conducted on selected variables with a *P*-value of less than 0.2. The final model was determined using variables with a significance level of 5%, and the model’s goodness of fit was evaluated using adjusted *R*^2^ values, considering a strong fit for adjusted *R*^2^ > 0.7[14]. Collinearity of the variables was checked. Linear regression models were formulated for models showing strong fit, and age and sex were included in the final model irrespective of their statistical significance. Statistical analysis was performed using Python 3.10.12.

### Categorization of age and preoperative WOMAC scores

We categorized age and preoperative WOMAC scores to investigate potential non-linear trends in the association between one-year WOMAC scores and ΔWOMAC scores. This approach aimed to identify the optimal age range and preoperative WOMAC score range, as multivariate regression assumes a linear relationship and may overlook non-linear associations. Age was categorized into four groups: below 60, 60–70, 70–80, and above 80 years. Preoperative WOMAC scores were classified based on quartile points, representing the lower 25%, median, and upper 25% of each score. Graphs were plotted to visualize curves, using the quartile boundaries as reference points for 1-year WOMAC scores and ΔWOMAC scores. The quartile points were 7, 9, and 11 for preoperative WOMAC pain; 2, 4, and 5 for preoperative WOMAC stiffness; and 26, 35, and 44 for preoperative WOMAC physical function scores. The differences between the categorized groups were analyzed using a one-way analysis of variance (ANOVA) test followed by Tukey’s HSD test as a post-hoc analysis.

## Results

Improvements were observed 1 year postoperatively in all subscales of the WOMAC. The pain score decreased from 9.3 preoperatively to 0.6, the stiffness score improved from 3.9 to 0.9, and the physical function score improved from 35.3 to 10.0. Collinearity was observed among the preoperative WOMAC pain, stiffness, and physical function scores. Consequently, the regression models for the 1-year and ΔWOMAC pain, stiffness, and physical function scores included only their corresponding preoperative scores as dependent variables.

### Factors affecting one-year WOMAC scores

The results of the multivariate regression analysis identifying factors influencing 1-year postoperative WOMAC scores are presented in Supplementary Tables 1–3. The regression models for 1-year WOMAC pain, stiffness, and physical function scores demonstrated poor goodness of fit, with adjusted *R*^2^ values of 0.017, 0.036, and 0.078, respectively.

### Factors affecting ΔWOMAC scores

The results of the multivariate regression analysis, identifying factors influencing the ΔWOMAC scores, are presented in Tables 3, 4 and 5. The regression models for ΔWOMAC pain, stiffness, and physical scores demonstrated strong goodness of fit, with adjusted *R*^2^ values of 0.881, 0.755, and 0.761, respectively.

**Table 3:** Regression analysis results of change ($\Delta )$ in WOMAC pain

	Univariate analysis			Multivariate analysis		
	Coefficient	*P*-value	95% CI	Coefficient	*P*-value	95% CI
Age (years)	0.035	0.013	[0.007, 0.062]	0.002	0.728	[−0.008, 0.011]
Sex (female)	−1.152	0.008	[−1.996, −0.307]	0.369	0.015*	[0.072, 0.665]
Body mass index	−0.038	0.209	[−0.097, 0.021]			
Preoperative HKAA	0.032	0.077	[−0.004, 0.068]	0.004	0.508	[−0.008, 0.017]
Postoperative HKAA	−0.018	0.716	[−0.112, 0.077]			
Cerebrovascular disease	0.005	0.987	[−0.634, 0.644]			
Diabetes mellitus	−0.205	0.363	[−0.646, 0.237]			
Hypertension	−0.002	0.994	[−0.404, 0.401]			
Ischemic heart disease	−0.500	0.122	[−1.132, 0.133]	−0.066	0.556	[−0.286, 0.154]
Chronic liver disease	−0.129	0.804	[−1.153, 0.895]			
Chronic kidney disease	0.312	0.572	[−0.770, 1.393]			
Patella resurfacing	−0.154	0.464	[−0.567, 0.259]			
Preoperative flexion contracture	−0.025	0.054	[−0.050, 0.000]	−0.003	0.524	[−0.012, 0.006]
Preoperative flexion angle	−0.001	0.854	[−0.013, 0.011]			
Postoperative flexion contracture	0.065	0.131	[−0.019, 0.149]	0.039	0.011*	[0.009, 0.070]
Postoperative flexion angle	−0.010	0.269	[−0.029, 0.008]			
Preoperative WOMAC pain	−0.975	0.000	[−0.998, −0.952]	−0.976	0.000*	[−0.999, −0.953]

Adjusted *R*^2^ = 0.881

$\boldsymbol{\Delta }$, Change in WOMAC scores; *C.I.* Confidence interval, *HKAA* Hip-knee-ankle angle, *WOMAC* Western Ontario and McMaster Universities Osteoarthritis Index

^*^statistically significant at *P* < 0.05

**Table 4:** Regression analysis results of change ($\Delta )$ in WOMAC stiffness

	Univariate analysis			Multivariate analysis		
	Coefficient	P-value	95% CI	Coefficient	*P*-value	95% CI
Age (years)	0.009	0.324	[−0.008, 0.025]	0.001	0.850	[−0.008, 0.009]
Sex (female)	−0.062	0.816	[−0.586, 0.462]	0.499	0.000*	[0.238, 0.760]
Body mass index	0.006	0.732	[−0.030, 0.043]			
Preoperative HKAA	0.003	0.804	[−0.019, 0.025]			
Postoperative HKAA	−004	0.894	[−0.062, 0.054]			
Cerebrovascular disease	0.123	0.541	[−0.272, 0.518]			
Diabetes mellitus	−0.205	0.140	[−0.478, 0.068]	0.040	0.574	[− 0.098, 0.178]
Hypertension	0.070	0.583	[−0.179, 0.318]			
Ischemic heart disease	−0.189	0.344	[−0.581, 0.202]			
Chronic liver disease	0.187	0.562	[−0.446, 0.820]			
Chronic kidney disease	−0.541	0.112	[−1.209, 0.127]	−0.188	0.279	[−0.527, 0.152]
Patella resurfacing	−0.021	0.872	[−0.276, 0.234]			
Preoperative flexion contracture	−0.019	0.015	[−0.035, −0.004]	−0.007	0.110	[−0.015, 0.002]
Preoperative flexion angle	−0.002	0.557	[−0.010, 0.005]	−0.001	0.537	[−0.006, 0.003]
Postoperative flexion contracture	−0.018	0.498	[−0.070, 0.034]			
Postoperative flexion angle	−0.010	0.078	[−0.022, 0.001]	−0.007	0.027*	[−0.014, −0.001]
Preoperative WOMAC stiffness	−0.942	0.000	[−0.976, −0.908]	−0.943	0.000*	[−0.977, −0.909]

Adjusted *R*^2^ = 0.755

$\boldsymbol{\Delta }$, Change in WOMAC scores; *C.I.* Confidence interval, *HKAA* Hip-knee-ankle angle, *WOMAC* Western Ontario and McMaster Universities Osteoarthritis Index

^*^statistically significant at *P* < 0.05

**Table 5:** Regression analysis results of change ($\Delta )$ in WOMAC physical function

	Univariate analysis			Multivariate analysis		
	Coefficient	*P*-value	95% CI	Coefficient	*P*-value	95% CI
Age (years)	0.109	0.043	[0.004, 0.215]	0.107	0.000*	[0.055, 0.160]
Sex (female)	−2.553	0.127	[−5.831, 0.726]	2.891	0.000*	[1.267, 4.515]
Body mass index	0.037	0.753	[−0.191, 0.265]			
Preoperative HKAA	0.123	0.081	[−0.015, 0.261]	0.018	0.611	[−0.050, 0.086]
Postoperative HKAA	0.056	0.763	[−0.309, 0.422]			
Cerebrovascular disease	1.724	0.172	[− 0.748, 4.195]	1.777	0.004*	[0.558, 2.996]
Diabetes mellitus	−1.272	0.144	[−2.980, 0.436]	−0.040	0.926	[−0.884, 0.805]
Hypertension	0.465	0.559	[− 1.094, 2.023]			
Ischemic heart disease	0.380	0.761	[−2.073, 2.833]			
Chronic liver disease	−0.831	0.681	[−4.794, 3.132]			
Chronic kidney disease	0.881	0.680	[−3.307, 5.068]			
Patella resurfacing	−3.987	0.000	[−5.566, −2.408]	−1.319	0.001*	[−2.123, −0.515]
Preoperative flexion contracture	−0.040	0.423	[−0.138, 0.058]			
Preoperative flexion angle	0.010	0.679	[−0.038, 0.058]			
Postoperative flexion contracture	0.237	0.155	[−0.090, 0.563]	0.179	0.037*	[0.010, 0.347]
Postoperative flexion angle	−0.061	0.092	[−0.133, 0.010]	−0.050	0.007*	[−0.087, −0.014]
Preoperative WOMAC physical function	−0.944	0.000	[−0.979, −0.910]	−0.945	0.000*	[−0.979, −0.910]

Adjusted *R*^2^ = 0.761

$\boldsymbol{\Delta }$, Change in WOMAC scores; *CI* confidence interval, *HKAA* hip-knee-ankle angle, *WOMAC* Western Ontario and McMaster Universities Osteoarthritis Index

^*^statistically significant at *P* < 0.05

Regarding ΔWOMAC pain scores, females and a higher degree of postoperative knee flexion contracture were associated with higher scores (*P* = 0.015 and *P* = 0.011), indicating a small clinical improvement. Conversely, higher preoperative WOMAC pain scores were related to lower scores (*P* = 0.000), indicating a large clinical improvement.

Regarding ΔWOMAC stiffness scores, females were associated with higher scores (*P* = 0.000), whereas higher postoperative knee flexion angle and higher preoperative WOMAC stiffness scores were related to lower scores (*P* = 0.027 and *P* = 0.000).

Regarding ΔWOMAC physical function scores, old age, females, cerebrovascular disease, and a higher degree of postoperative knee flexion contracture were associated with higher scores (*P* = 0.000, *P* = 0.000, *P* = 0.004, and *P* = 0.037, respectively). In contrast, patellar resurfacing, higher postoperative knee flexion angle, and higher preoperative WOMAC physical function scores were related to lower scores (*P* = 0.001, *P* = 0.007, and *P* = 0.000, respectively).

As the adjusted *R*^2^ was above 0.7 for all ΔWOMAC score models, the following regression equations were generated:

**ΔWOMAC pain** = −0.095 + 0.002 × (*age*) + 0.369 × (*female* = *1*) + 0.039 × (*postoperative flexion contracture*)—0.976 × (*preoperative WOMAC pain*).

**ΔWOMAC stiffness** = 1.289 + 0.001 × (*age*) + 0.499 × (*female* = *1*)—0.007 × (*postoperative flexion angle*)—0.943 × (*preoperative WOMAC stiffness*).

**ΔWOMAC physical function** = 4.196 + 0.107 × (*age*) + 2.891 × (*female* = *1*) + 1.777 × (*cerebrovascular disease* = *1*)—1.319 × (*patellar resurfacing* = *1*) + 0.179 × (*postoperative flexion contracture*)—0.050 × (*postoperative flexion angle*)—0.945 × (*preoperative WOMAC physical function*).

The scores obtained from the regression equation represent the predicted postoperative WOMAC scores, calculated on the basis of preoperative key variables.

### Categorization of age and preoperative WOMAC scores

**Fig. 2:**
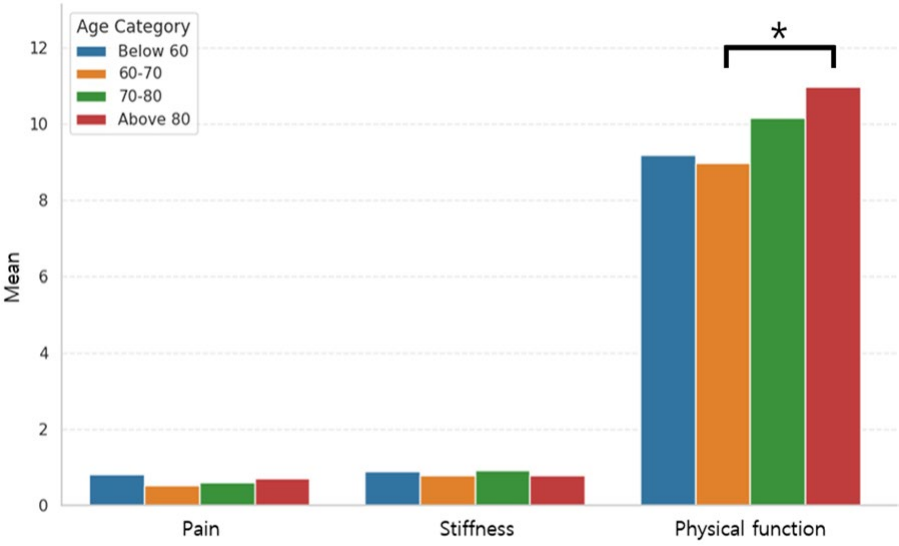
The 1-year WOMAC scores according to age category. *statistically significant at *P* < 0.05. *WOMAC* Western Ontario and McMaster Universities Osteoarthritis Index

**Fig. 3:**
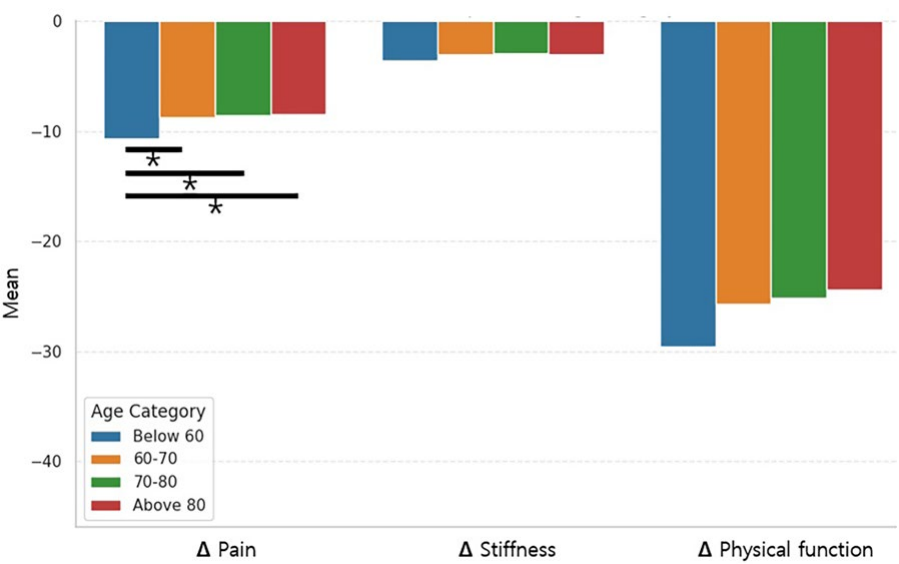
Change (Δ) in WOMAC scores according to age category. *statistically significant at *P* < 0.05. *WOMAC* Western Ontario and McMaster Universities Osteoarthritis Index

Regarding age, physical function at 1 year was best in the 60 s and was poorest in patients above aged 80 years. The 1-year pain and stiffness scores were not significantly different between the quartile groups, and no trend was seen (Fig. 2). Patients below age 60 showed the most improvement in pain, and the amount of improvement decreased with increasing age (*P* < 0.05). Changes in stiffness and physical function also showed a similar trend, although not statistically significant (Fig. 3).

**Fig. 4:**
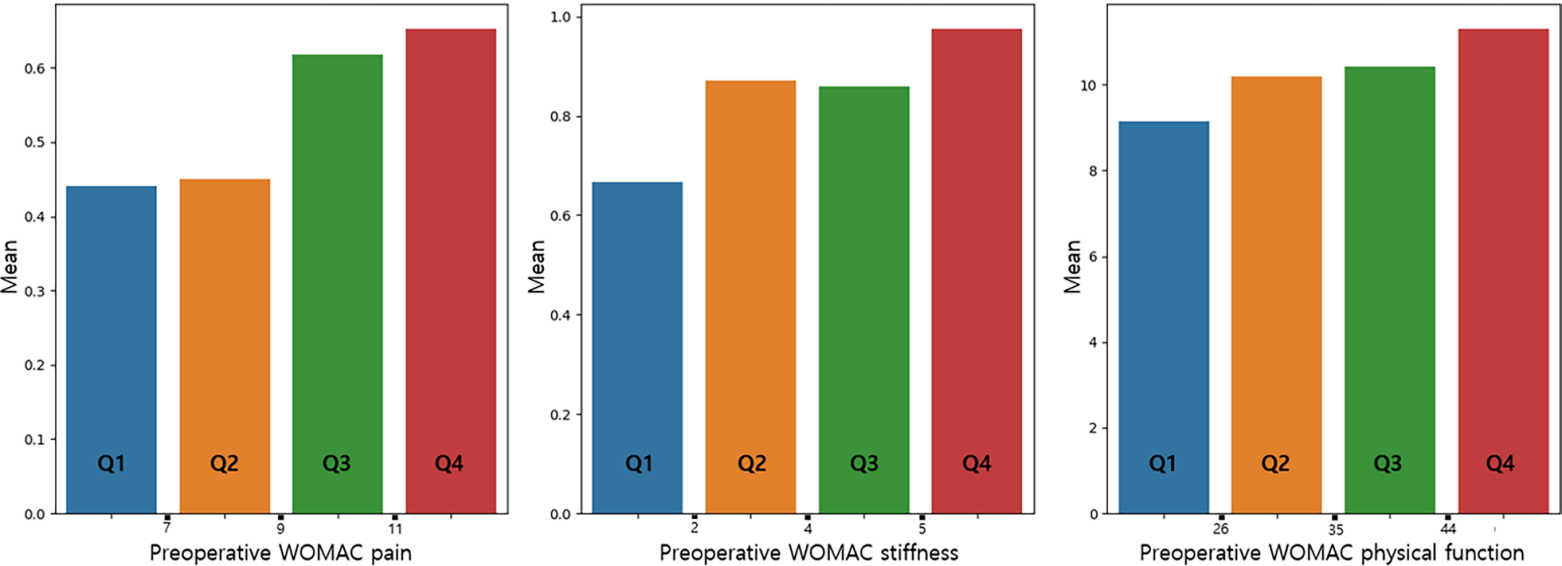
The 1-year WOMAC scores according to preoperative WOMAC scores. There were no significant differences between the quartile groups in each WOMAC scores. *WOMAC* Western Ontario and McMaster Universities Osteoarthritis Index

**Fig. 5:**
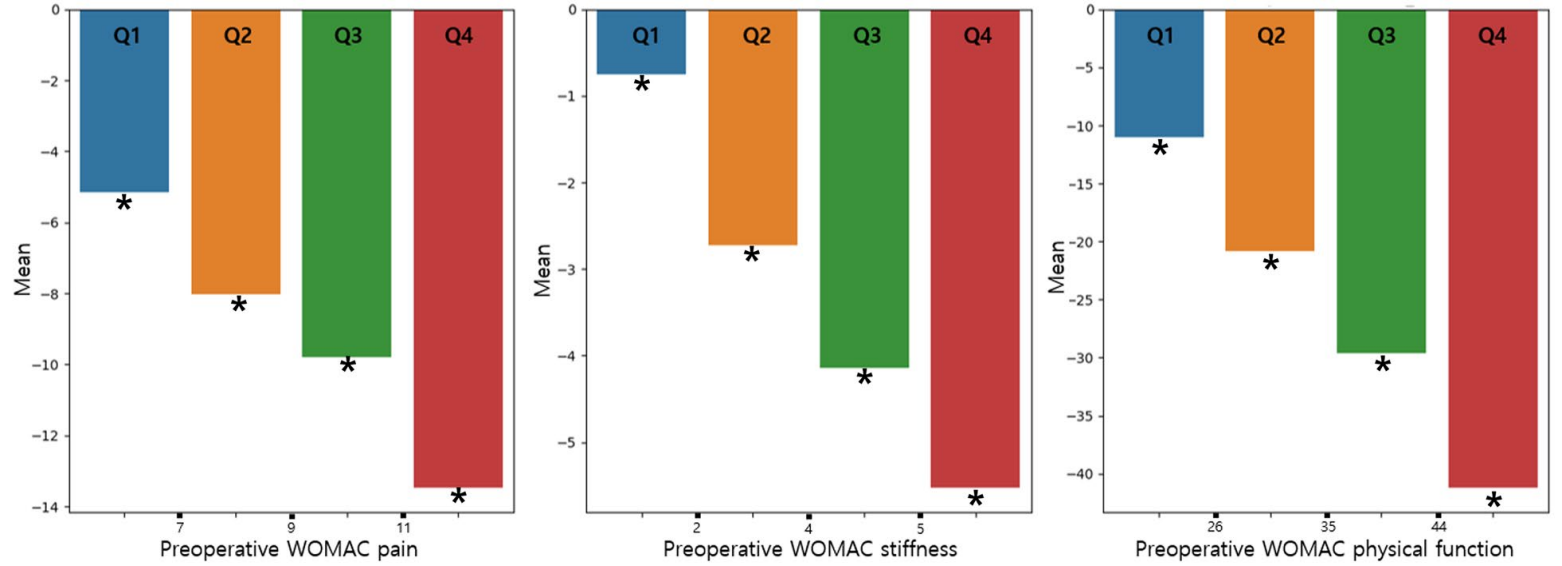
Change (Δ) in WOMAC scores according to preoperative WOMAC scores. All quartile groups in each WOMAC scores are statistically different (*P* < 0.05) from one another (one-way analyses of variance test followed by Tukey’s HSD post hoc analyses). *WOMAC* Western Ontario and McMaster Universities Osteoarthritis Index

Figures 4 and 5 demonstrate the one-year WOMAC and ΔWOMAC scores according to preoperative WOMAC categories. One-year WOMAC scores showed an increasing trend as the scores of the quartile groups increased, consistent with the multivariate regression analysis. In contrast, ΔWOMAC scores displayed a decreasing trend as preoperative WOMAC stiffness increased, with statistically significant differences between each quartile group.

## Discussion

The most important finding of this study was the strong predictive correlation between perioperative factors and changes in WOMAC scores after TKA. Preoperative WOMAC scores, sex, and postoperative knee ROM significantly influenced the amount of change in all WOMAC scores. Additionally, age, cerebrovascular disease, and patellar resurfacing were related to ΔWOMAC physical function scores. The strength of our study lies in its ability to provide preoperative patient counseling, as it could predict the extent of improvements in WOMAC scores after surgery.

Previous studies addressed the optimal TKA outcomes, which can be divided into two aspects: obtaining the maximum clinical score value itself after surgery and achieving the greatest clinical improvement [6]. Prior research generally supported an association between better preoperative PROMs and improved postoperative PROMs [15, 16]. Meanwhile, poor preoperative PROMs predicted greater improvement. Vina et al. [17] found that patients with improvements after TKA had higher preoperative WOMAC scores, even if their postoperative results were not as good as those with better preoperative WOMAC scores. Thus, both postoperative absolute values and changes in PROMs should be considered. Meanwhile, although the definition of an improved outcome varies in the literature, previous research reported a minimum clinically important difference (MCID) for WOMAC total scores (the sum of pain, stiffness, and physical function scores) of 10 to 15 [18, 19]. While the analysis of MCID was not conducted in this study, the results demonstrated satisfactory outcomes that exceeded the MCID threshold.

The predictive accuracy of the model for 1-year WOMAC scores was found to be low, whereas the model for predicting changes in WOMAC scores demonstrated superior predictive accuracy. This discrepancy can be attributed to the phenomenon where the absolute postoperative WOMAC scores converged, as there were greater clinical improvements in the group with poor preoperative WOMAC scores. As a result, there was no substantial difference between the maximum postoperative PROM values in patients with poor preoperative PROMs and those with good preoperative PROMs. Interestingly, the 1-year WOMAC pain scores were not affected by preoperative WOMAC pain scores, possibly owing to the strong pain-relieving effect of TKA, resulting in low pain levels. However, the higher predictive power of ΔWOMAC scores can be explained by the diverse scattering of preoperative WOMAC scores.

Sex was a significant factor affecting all WOMAC scores, with inferior 1-year outcomes and changes observed in females compared to males. While the impact of sex on TKA outcomes may vary, it is generally accepted that women experience greater pain and functional impairment compared to men prior to TKA [20, 21]. A study reporting inferior TKA results for women suggested that women had more symptomatic joints than men, in which WOMAC scores were affected not only by the knee but by the overall condition [20]. In addition, according to Lingard et al. [22], women had a higher level of depression compared to men, which may have a greater impact on physical impairment after TKA.

Measures of knee ROM, such as flexion contracture and flexion angle, had an impact on all ΔWOMAC scores, one-year WOMAC stiffness and physical function scores. Patients with a large degree of residual flexion contracture reported little change in pain and function, whereas patients with higher postoperative flexion angles exhibited more improvement in stiffness and function. As this study was conducted on an East Asian population, it is important to note that cultural practices often involve spending time on the floor, including activities, such as kneeling and squatting for daily chores. In this cultural context, a lower flexion angle after TKA may result in a perceived sense of stiffness. To comfortably perform activities listed in the WOMAC physical function questionnaire, such as rising from bed, sitting, and engaging in light domestic duties, patients in this population may require higher flexion angles. Ritter et al. [23] reported that flexion contractures remaining after TKA deteriorated pain and function, as well as patient satisfaction. Similarly, Kubo et al. [24] reported that knee function was affected by the postoperative flexion angle after TKA.

Age was found to be a significant factor influencing 1-year and ΔWOMAC physical function scores after TKA. Advancing age was associated with inferior WOMAC scores, suggesting a negative impact of aging on functional outcomes after surgery. Interestingly, an age range of 60–70 years was identified as the period with maximal one-year physical function, which was visualized graphically (Fig. 2). Studies regarding age and PROMs have reported conflicting findings, with some showing no association, and others reporting older age as a predictor of poorer PROMs [15, 25, 26]. Results comparable to ours include those of Chang et al., who reported poorer WOMAC scores with increasing age [26] and Lee et al., who reported that optimal PROMs were achieved in patients between 70 and 80 years, with the best outcomes observed around 70 years [5].

Cerebrovascular disease was the only comorbidity significantly affecting 1-year and ΔWOMAC scores, particularly in physical function. A study by Singh et al. [27] based on a United States joint registry reported poor outcomes for patients with cerebrovascular disease, and recommended sharing the risk of poor functional outcomes with patients with cerebrovascular disease. Pomeroy et al. [28] reported that patients with neurologic disorders may present challenging conditions, including contractures, muscle weakness, and instability, affecting recovery. However, improvements in functional outcomes were still observed. With the expanding indications for TKA, patients with cerebrovascular disease may benefit from TKA with advances in perioperative management, and the results of this study could aid in decision-making for these patients.

Patellar resurfacing also influenced outcomes after TKA. Patients with patellar resurfacing showed worse 1-year WOMAC stiffness scores but better 1-year WOMAC scores and improved ΔWOMAC physical function scores. Some meta-analyses reported no differences between patellar resurfacing and non-resurfacing, except for secondary resurfacing rates, whereas some studies reported better functional results with patellar resurfacing [29, 30]. However, studies comparing stiffness after patellar resurfacing or non-resurfacing are limited. In addition, the WOMAC stiffness score is not always indicative of restricted knee ROM. Rather, it relates to initiating movement after prolonged inactivity instead of knee ROM [31]. Nevertheless, this study demonstrated a significant difference between patellar resurfacing and non-resurfacing groups in terms of stiffness and physical function, adding to the knowledge of patella management.

There were some limitations to this study. First, it had a relatively short follow-up period of only 1 year to assess clinical outcomes. Additionally, the regression model for 1-year WOMAC scores demonstrated poor predictability. In contrast, the predictability for ΔWOMAC scores was good. This could be because the study was based on the results of TKA performed at a single center, with the use of posterior stabilized and fixed implants. While this aspect may serve as a good controlling factor, its generalizability to other patients using different implants is limited. Furthermore, the absence of an analysis for the MCID is a limitation, as such analysis could provide further insights into clinically meaningful improvements. Lastly, the high proportion of female patients (over 90%), which reflects the demographic trends in South Korea where approximately 90% of TKAs are performed on women, may have influenced the study outcomes [32].

## Conclusions

A quantitative model was developed to predict changes in postoperative WOMAC scores following TKA, demonstrating strong predictability. Preoperative WOMAC scores, sex, and postoperative knee ROM were identified as significant factors influencing all aspects of pain, stiffness, and physical function in the WOMAC assessment. Age, cerebrovascular disease, and patellar resurfacing were found to be associated with changes in physical function.

## Supplementary Information

Supplementary material 1: Table 1. Regression analysis results of one-year WOMAC pain. Table 2. Regression analysis results of one-year WOMAC stiffness. Table 3. Regression analysis results of one-year WOMAC physical function.

## Data Availability

Data available on request from the authors.
