# Supplementary material for: Can individual functional improvements be predicted in osteoarthritic patients after total knee arthroplasty?
Source: Knee Surg Relat Res. 2024 Oct 14;36:31. doi: 10.1186/s43019-024-00238-1 (PMC11479557; doi:10.1186/s43019-024-00238-1)
Supplement: Supplementary file 1 — Supplementary material 1: Table 1. Regression analysis results of one-year WOMAC pain. Table 2. Regression analysis results of one-year WOMAC stiffness. Table 3. Regression analysis results of one-year WOMAC physical function. [file 43019_2024_238_MOESM1_ESM.docx]

Supplementary table 1. Regression analysis results of one-year WOMAC pain.

|  | **Univariate analysis** | | | **Multivariate analysis** | | |
| --- | --- | --- | --- | --- | --- | --- |
|  | Coefficient | P-value | 95% C.I. | Coefficient | P-value | 95% C.I. |
| Age (years) | 0.002 | 0.621 | [-0.007, 0.012] | 0.001 | 0.795 | [-0.008, 0.011] |
| Sex (female) | 0.401 | 0.007 | [0.108, 0.694] | 0.388 | 0.010* | [0.093, 0.682] |
| Body mass index | -0.002 | 0.846 | [-0.022, 0.018] |  |  |  |
| Preoperative HKAA | 0.002 | 0.692 | [-0.010, 0.015] |  |  |  |
| Postoperative HKAA | 0.018 | 0.270 | [-0.014, 0.051] |  |  |  |
| Cerebrovascular disease | 0.185 | 0.101 | [-0.036, 0.406] | 0.141 | 0.213 | [-0.081, 0.362] |
| Diabetes mellitus | 0.082 | 0.292 | [-0.071, 0.235] |  |  |  |
| Hypertension | 0.097 | 0.175 | [-0.043, 0.236] | 0.054 | 0.450 | [-0.086, 0.194] |
| Ischemic heart disease | -0.077 | 0.490 | [-0.297, 0.142] |  |  |  |
| Chronic liver disease | -0.287 | 0.113 | [-0.641, 0.068] | -0.199 | 0.275 | [-0.556, 0.158] |
| Chronic kidney disease | 0.197 | 0.302 | [-0.178, 0.572] |  |  |  |
| Patella resurfacing | 0.146 | 0.045 | [0.003, 0.289] | 0.136 | 0.066 | [-0.009, 0.281] |
| Preoperative flexion contracture | 0.001 | 0.870 | [-0.008, 0.010] |  |  |  |
| Preoperative flexion angle | -0.002 | 0.345 | [-0.006, 0.002] |  |  |  |
| Postoperative flexion contracture | 0.034 | 0.021 | [0.005, 0.064] | 0.026 | 0.097 | [-0.005, 0.056] |
| Postoperative flexion angle | -0.005 | 0.113 | [-0.012, 0.001] | -0.004 | 0.190 | [-0.011, 0.002] |
| Preoperative WOMAC pain | 0.025 | 0.030 | [0.002, 0.048] | 0.021 | 0.071 | [-0.002, 0.044] |

*statistically significant at P < 0.05

Adjusted R² = 0.017
C.I., Confidence interval; HKAA, Hip-knee-ankle angle; WOMAC, The Western Ontario and McMaster Universities Osteoarthritis Index

Supplementary table 2. Regression analysis results of one-year WOMAC stiffness.

|  | **Univariate analysis** | | | **Multivariate analysis** | | |
| --- | --- | --- | --- | --- | --- | --- |
|  | Coefficient | P-value | 95% C.I. | Coefficient | P-value | 95% C.I. |
| Age (years) | -0.000 | 0.976 | [-0.009, 0.008] | -0.001 | 0.840 | [-0.009, 0.008] |
| Sex (female) | 0.511 | 0.000 | [0.251, 0.772] | 0.516 | 0.000* | [0.256, 0.775] |
| Body mass index | -0.009 | 0.349 | [-0.027, 0.010] |  |  |  |
| Preoperative HKAA | -0.009 | 0.129 | [-0.020, 0.002] | -0.008 | 0.132 | [-0.019, 0.003] |
| Postoperative HKAA | -0.005 | 0.759 | [-0.034, 0.025] |  |  |  |
| Cerebrovascular disease | 0.092 | 0.362 | [-0.106, 0.290] |  |  |  |
| Diabetes mellitus | 0.034 | 0.626 | [-0.103, 0.171] |  |  |  |
| Hypertension | 0.116 | 0.068 | [-0.008, 0.241] | 0.086 | 0.173 | [-0.038, 0.209] |
| Ischemic heart disease | -0.095 | 0.341 | [-0.292, 0.101] |  |  |  |
| Chronic liver disease | -0.104 | 0.520 | [-0.421, 0.213] |  |  |  |
| Chronic kidney disease | -0.195 | 0.254 | [-0.530, 0.140] |  |  |  |
| Patella resurfacing | 0.173 | 0.008 | [0.046, 0.301] | 0.187 | 0.005* | [0.058, 0.316] |
| Preoperative flexion contracture | -0.002 | 0.634 | [-0.010, 0.006] |  |  |  |
| Preoperative flexion angle | -0.003 | 0.189 | [-0.006, 0.001] | 0.000 | 0.953 | [-0.004, 0.005] |
| Postoperative flexion contracture | 0.012 | 0.349 | [-0.014, 0.039] |  |  |  |
| Postoperative flexion angle | -0.007 | 0.022 | [-0.012, -0.001] | -0.008 | 0.014* | [-0.015, -0.002] |
| Preoperative WOMAC stiffness | 0.058 | 0.001 | [0.024, 0.092] | 0.050 | 0.004* | [0.016, 0.084] |

*statistically significant at P < 0.05

Adjusted R² = 0.036
C.I., Confidence interval; HKAA, Hip-knee-ankle angle; WOMAC, The Western Ontario and McMaster Universities Osteoarthritis Index

Supplementary table 3. Regression analysis results of one-year WOMAC physical function.

|  | **Univariate analysis** | | | **Multivariate analysis** | | |
| --- | --- | --- | --- | --- | --- | --- |
|  | Coefficient | P-value | 95% C.I. | Coefficient | P-value | 95% C.I. |
| Age (years) | 0.111 | 0.000 | [0.058, 0.164] | 0.104 | 0.000* | [0.052, 0.157] |
| Sex (female) | 3.079 | 0.000 | [1.430, 4.729] | 2.810 | 0.001* | [1.195, 4.426] |
| Body mass index | -0.091 | 0.121 | [-0.206, 0.024] | -0.095 | 0.105 | [-0.211, 0.020] |
| Preoperative HKAA | 0.006 | 0.864 | [-0.064, 0.076] |  |  |  |
| Postoperative HKAA | -0.021 | 0.826 | [-0.206, 0.164] |  |  |  |
| Cerebrovascular disease | 1.822 | 0.004 | [0.575, 3.069] | 1.694 | 0.006* | [0.483, 2.905] |
| Diabetes mellitus | 0.169 | 0.702 | [-0.696, 1.034] |  |  |  |
| Hypertension | 0.752 | 0.061 | [-0.035, 1.539] | 0.641 | 0.107 | [-0.139, 1.420] |
| Ischemic heart disease | -0.719 | 0.255 | [-1.960, 0.521] |  |  |  |
| Chronic liver disease | -1.868 | 0.067 | [-3.870, 0.134] | -1.282 | 0.200 | [-3.241, 0.678] |
| Chronic kidney disease | 0.677 | 0.531 | [-1.442, 2.795] |  |  |  |
| Patella resurfacing | -1.112 | 0.007 | [-1.918, -0.306] | -1.473 | 0.000* | [-2.288, -0.658] |
| Preoperative flexion contracture | 0.105 | 0.000 | [0.056, 0.154] | 0.078 | 0.003* | [0.026, 0.129] |
| Preoperative flexion angle | -0.031 | 0.012 | [-0.055, -0.007] | -0.015 | 0.290 | [-0.043, 0.013] |
| Postoperative flexion contracture | 0.258 | 0.002 | [0.093, 0.422] | 0.115 | 0.184 | [-0.055, 0.284] |
| Postoperative flexion angle | -0.067 | 0.000 | [-0.103, -0.031] | -0.029 | 0.179 | [-0.070, 0.013] |
| Preoperative WOMAC physical function | 0.056 | 0.002 | [0.021, 0.090] | 0.047 | 0.007* | [0.013, 0.081] |

*statistically significant at P < 0.05

Adjusted R² = 0.078
C.I., Confidence interval; HKAA, Hip-knee-ankle angle; WOMAC, The Western Ontario and McMaster Universities Osteoarthritis Index
